# Repeatability of quantitative individual lesion and total disease multiparametric whole-body MRI measurements in prostate cancer bone metastases

**DOI:** 10.1259/bjr.20230378

**Published:** 2023-09-03

**Authors:** Ricardo Donners, Antonio Candito, Matthew Blackledge, Mihaela Rata, Christina Messiou, Dow-Mu Koh, Nina Tunariu

**Affiliations:** 1 Department of Radiology, Royal Marsden Hospital, Sutton, United Kingdom; 2 Department of Radiology, University Hospital Basel, Basel, Switzerland; 3 Cancer Research UK Cancer Imaging Centre, The Institute of Cancer Research, Sutton, United Kingdom

## Abstract

**Objectives:**

To assess the repeatability of quantitative multiparametric whole-body MRI (mpWB-MRI) parameters in advanced prostate cancer (APC) bone metastases.

**Methods:**

1.5T MRI was performed twice on the same day in 10 APC patients. MpWB-MRI-included diffusion weighted imaging (DWI) and *T*_1_-weighted gradient-echo 2-point Dixon sequences. ADC and relative fat-fraction percentage (rFF%) maps were calculated, respectively. A radiologist delineated up to 10 target bone metastases per study. Means of ADC, b900 signal intensity(SI), normalised b900 SI, rFF% and maximum diameter (MD) for each target lesion and overall parameter averages across all targets per patient were recorded. The total disease volume (tDV in ml) was manually delineated on b900 images and mean global (g)ADC was derived. Bland-Altman analyses were performed with calculation of 95% repeatability coefficients (RC).

**Results:**

Seventy-three individual targets (median MD 26 mm) were included. Lesion mean ADC RC was 12.5%, mean b900 SI RC 137%, normalised mean b900 SI RC 110%, rFF% RC 3.2 and target MD RC 5.5 mm (16.3%). Patient target lesion average mean ADC RC was 6.4%, b900 SI RC 104% and normalised mean b900 SI RC 39.6%. Target average rFF% RC was 1.8, average MD RC 1.3 mm (4.8%). tDV segmentation RC was 6.4% and mean gADC RC 5.3%.

**Conclusions:**

APC bone metastases’ ADC, rFF% and maximum diameter, tDV and gADC show good repeatability.

**Advances in knowledge:**

APC bone metastases’ mean ADC and rFF% measurements of single lesions and global disease volumes are repeatable, supporting their potential role as quantitative biomarkers in metastatic bone disease.

## Introduction

Ninety percent of advanced prostate cancer (APC) patients develop bone metastases and in up to 45% the skeleton is the only metastatic site.^1^ However, conventional imaging including CT and anatomic MRI is inadequate for response assessment of bone disease.^2,3^

Multiparametric whole-body MRI (mpWB-MRI) including DWI and Dixon sequences has emerged as a promising imaging biomarker for APC bone metastases’ therapy response.^4^ DWI and Dixon are non-invasive techniques that provide information on tissue composition without the need for ionising radiation, tracer or contrast application. While the former informs on tissue water mobility and tumour cellularity, the latter can provide information on relative tissue fat content. Both techniques facilitate identification, staging and response assessment of bone metastases and allow for quantitative measurements.^4–11^

The apparent diffusion coefficient (ADC) is the most established quantitative DWI parameter and most commonly calculated from mono-exponential modelling of the signal intensity decay between high b-value (strong diffusion-weighting) and low b-value (weak diffusion-weighting) DWI. ADC has been shown to inversely correlate with lesion cellularity in multiple cancers, including prostate cancer bone metastases.^5,12–16^ An increase in ADC following treatment correlates with reduction in tumour cellularity and favourable therapy results.^4^ Consequently, ADC was suggested as a quantitative imaging biomarker in bone disease, with growing evidence for its clinical and biological validation.^5,6^ Clinically employed, fast 2-point Dixon MRI allows for calculation of relative fat-fraction percentages (rFF%). RFF% was shown to allow for differentiation between benign and malignant bone marrow conditions and inversely correlated with APC bone metastases’ cellularity.^5,17,18^

Despite its quantitative potential, the mainstay of mpWB-MRI bone assessment in cancer patients is confined to visual/semi-quantitative interpretation of the DWI, ADC and Dixon images.^11^ Quantitative evaluation is mostly limited to research applications.^4,19,20^ One major impediment of wider translation of ADC and other quantitative imaging biomarkers, such as rFF%, into clinical practice is the limited evidence of technical validation.^19^ Assessment of measurement precision and consistency by analysing repeatability documents the ability of a measurement to be duplicated. Repeatability can be described via several descriptors, including limits of agreement (LoA) and repeatability coefficients (RCs). LoA and RCs are relevant as they describe thresholds for parameter measurement differences to be meaningful, representing true biological change rather than measurement error, biological or technical variability.^19^ Thus, knowledge of LoA or RCs is crucial for determining quantitative biomarker thresholds for disease identification and response assessment. Recent studies showed good ADC test-retest repeatability in monoclonal plasma cell disorders,^21,22^ but data for focal metastatic bone disease are lacking.

Another factor contributing to the translational gap between mpWB-MRI biomarker research and clinical application is the reliance on advanced software for metastatic disease segmentation in imaging biomarker studies.^7,23^ By contrast, quantitative assessments in clinical practice are mostly confined to region of interest (ROI) measurements on PACS workstations. Software development aiming towards clinical application for facilitated disease segmentation is ongoing. In the meantime, pragmatic quantitative bone disease measurements with immediate clinical utility, similar to the widely adopted RECIST 1.1, are desirable.^24^

The aim of this study was to assess the repeatability of the quantitative mpWB-MRI parameters ADC and rFF% in APC bone metastases derived from pragmatic/real life target lesion ROI measurements in comparison with total malignant disease segmentation.

## Methods and materials

### Study design

This prospective repeatability study was approved by the local research and ethics committee. APC patients were recruited in one institution and informed consent was obtained from each participant. Study inclusion criteria were histopathology diagnosis of prostate cancer, history of bone metastases, and no contraindication for MRI acquisition. Exclusion criteria were contraindications for MRI acquisition. Eventually, 11 prostate cancer patients with a median age of 67.5 years were recruited.

### Imaging acquisition

Initial and repeat mpWB-MRI were acquired between 2013-08-02 and 201311-08 on Siemens MAGNETOM Aera 1.5T MRI (Siemens Healthineers, Erlangen, Germany). Patients were scanned twice in one setting, with repositioning between the examinations. Median time interval between initial and re-test imaging sequences was 54 min.

The imaging protocol including parameters for DWI and CAIPIRINHA (Controlled Aliasing in Parallel Imaging Results in Higher Acceleration) accelerated *T*_1_-weighted Dixon MRI is shown in Table 1.

**Table 1.** MRI acquisition parameters

Parameter	DWI	*T*_1_-weighted GE DIXON
b-values in s/mm^2^	b50, b600 b900	-
TE in ms	69	2.39
TR in ms	11300	7.63
Slice thickness in mm	6	5
Fat-suppression	Short Tau Inversion Recovery	DIXON
Averages	3 (b50), 5 (b600), 5 (b900)	1
Slice spacing in mm	6	6
Pixel bandwidth in Hz	1955	400
Acquisition Matrix in pixels	128*104	256*156
Image matrix in pixels	256*208	256*208
Flip angle	90°	10°
Gradient direction	three orthogonal (trace)	-

DWI, diffusion weighted MRI; GE, gradient-echo.

### Image analysis

Imaging analysis was performed in two ways. The first approach aimed to mimic clinical practise. Measurements were performed on PACS (IDS7, Sectra AB, Sweden) available on reporting workstations in the department.

**Figure 1.**
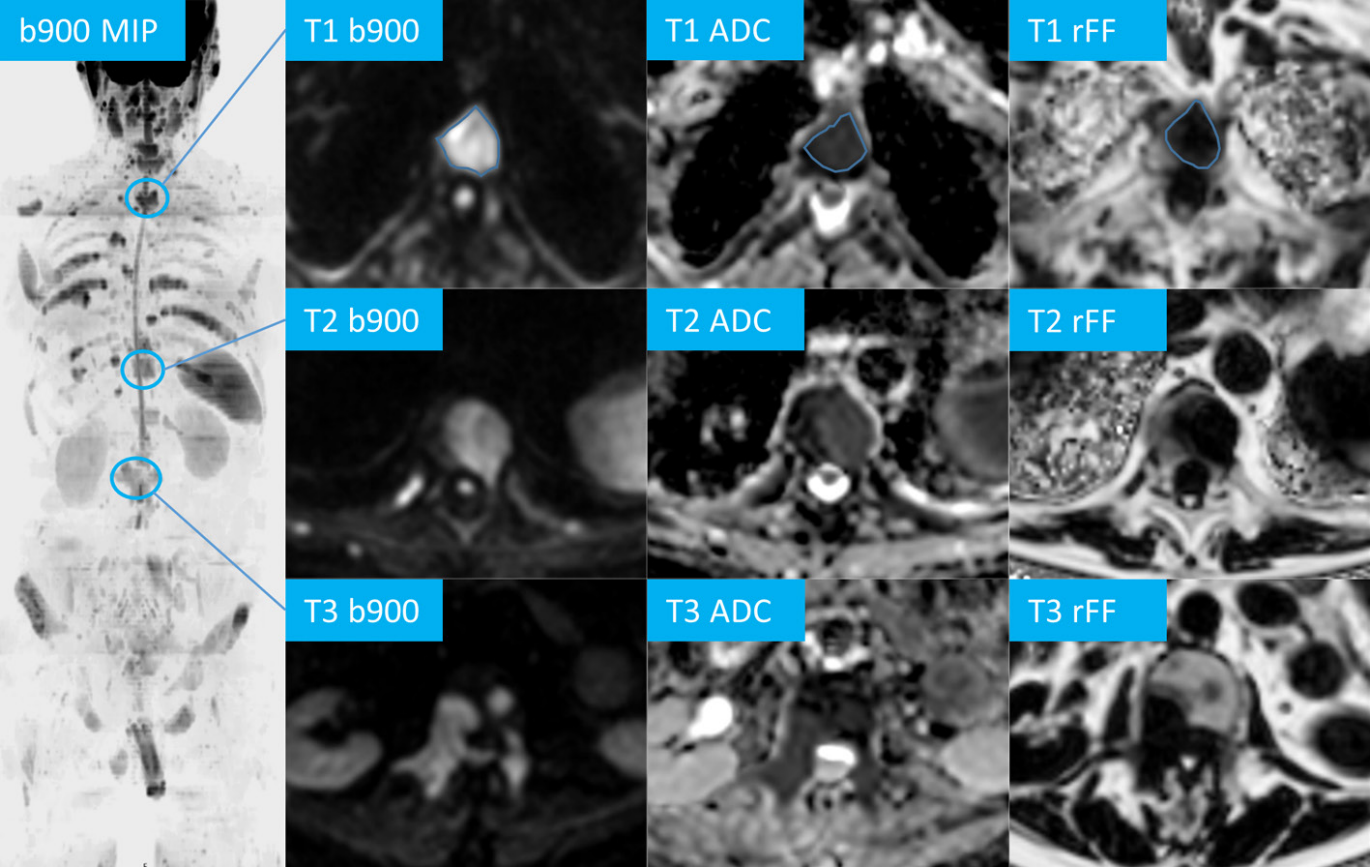
Target lesion measurements in a 72-year-old prostate cancer patient, coronal maximum intensity projection (MIP) of the axial b900 DW images, three example targets (T1 – 3) of vertebral bodies on b900, ADC and relative fat-fraction (rFF) images, region of interest free-hand region of interest measurements are exemplified for T1.

Clinical imaging analyses were performed by a dedicated radiology fellow with four years of experience in functional imaging of malignant bone disease. In each patient, up to 10 bone metastases were chosen as target lesions. Measuring 10 lesions is assumed to be able to reflect on heterogeneous response in future applications. The importance of heterogeneous response was acknowledged by the Protate Cancer Working Group (PCWG), increasing the number of possible targets per organ in PCWG3 compared with PCWG2 criteria.^2^ In the present study, a suitable target bone metastasis was defined as a focal lesion with low signal on rFF% images compared to adjacent bone marrow, unsuppressed and consecutive high signal on b50 and b900 DWI and mean ADC < 1400 µm^2^/s. Lesions with mean ADC > 1400 µm^2^/s were evaluated on previous imaging when available and were suitable for inclusion when they showed unequivocal change in size and/or in ADC ≥ 30% compared with previous mpWB-MRI, identifying them as bone metastases.^4^ Similar to the target lesion selection approach described for RECIST 1.1 in soft-tissues, targets measured >1 cm in axial planes and larger, representative lesions were favoured.^24^ Using the PACS free-hand region of interest (ROI) tool, one metastasis was outlined on three consecutive slice ROIs on each of the b900, ADC and rFF% images, respectively. The average mean ADC, b900 SI and rFF% value across the three ROIs was documented for all target lesions (Figure 1). Additionally, three consecutive ROIs were placed in the conus medullaris on b900 images and the average mean SI was recorded. Normalised target lesion b900 SI values were calculated by division of lesion b900 SI by the conus medullaris b900 mean SI. The maximum diameter of each target lesion was measured on rFF% images.

**Figure 2.**
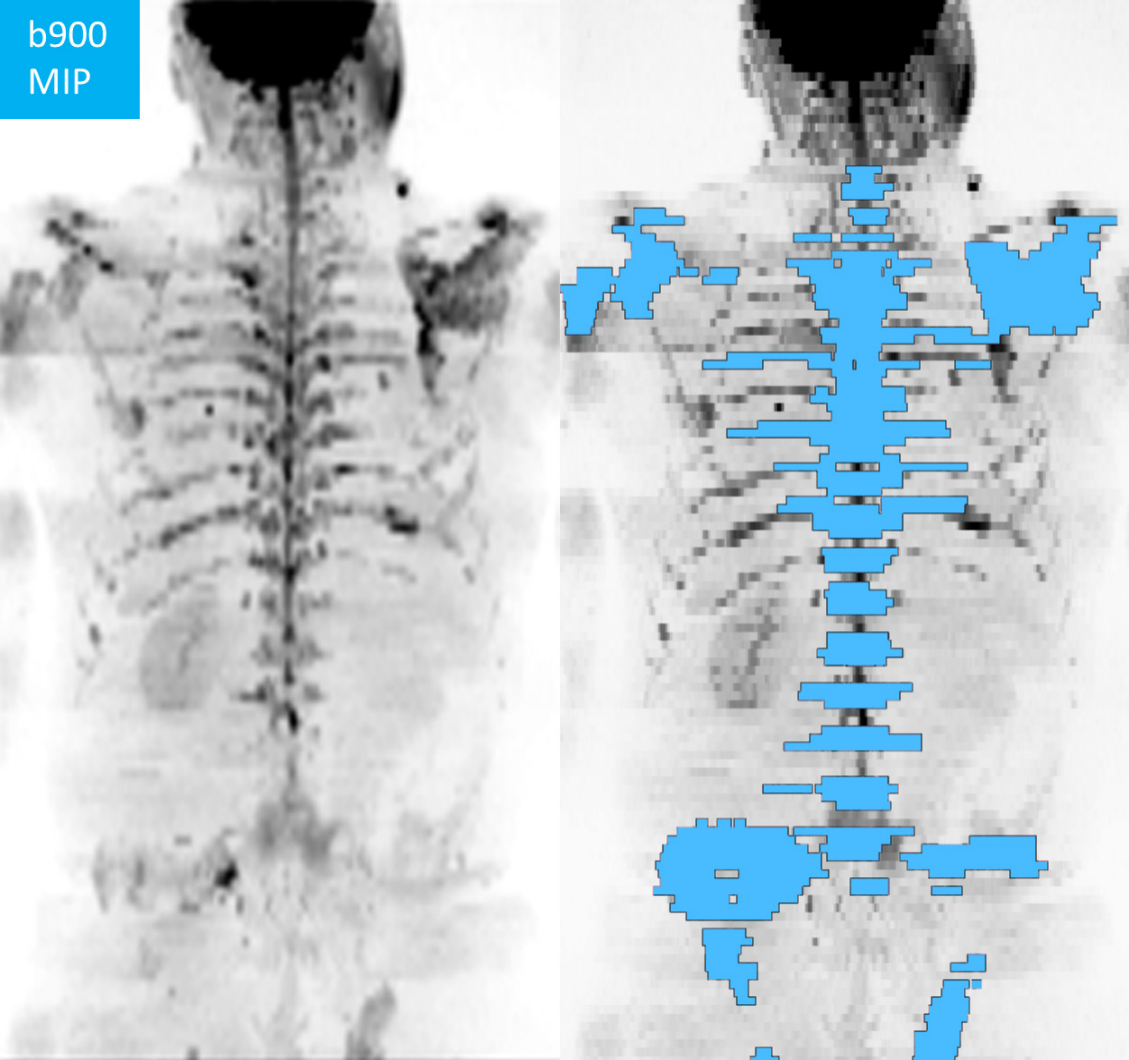
Total bone disease volume segmentation in a 61-year-old metastatic prostate cancer patient, DWI b900 maximum intensity projection (MIP) with superimposed disease volumes of interest (blue).

A second, volumetric analysis was performed by a board-certified consultant radiologist, with 15 years of experience in mpWB-MRI interpretation, using commercially available postprocessing software (OsiriX, version 56, PixmeoSARL Bernex, Switzerland). All metastases, identified using all available sequences as described above, were segmented on b900 images for each patient (Figure 2). The generated volumes of interest (VOIs) encompassing the total disease volume (tDV in ml) were copied from b900 images onto corresponding ADC maps. From the tDV segmentation, global ADC (gADC) mean and median values were derived for each patient.

### Statistical analyses

Statistical analysis was performed using commercially available software (IBM SPSS Statistics Version 25, IBM Corp. Armonk, New York, USA). Individual target lesion ROI means of ADC, b900 SI, normalised b900 SI, rFF% and maximum diameter, per-patient parameter averages across all target lesions and volumetric segmentation derived parameter repeatability was assessed using the methodology described by Bland and Altman.^25^ Upper and lower 95% LoA were derived. RCs were calculated according to the methodology suggested by Beckermann et al.^26^ The Wilcoxon signed-rank test was used for paired comparison of patient average mean ADC from target lesions and gADC measurements. A *p*-value < 0.05 was deemed significant.

## Results

One patient did not show bone metastases on mpWB-MRI; hence, 10 patients with a median age of 67.5 years and a median Gleason score of 4 + 4 were included for final analyses. All patients were metastatic castrate-resistant and had undergone all lines of standard of care treatment. At the time of the study, all patients were undergoing systemic anticancer therapy and no patient was chemotherapy-naïve. In total, 73 target lesions were chosen and delineated across all subjects. tDV segmentation was successful in all patients.

### Individual target lesions

**Figure 3.**
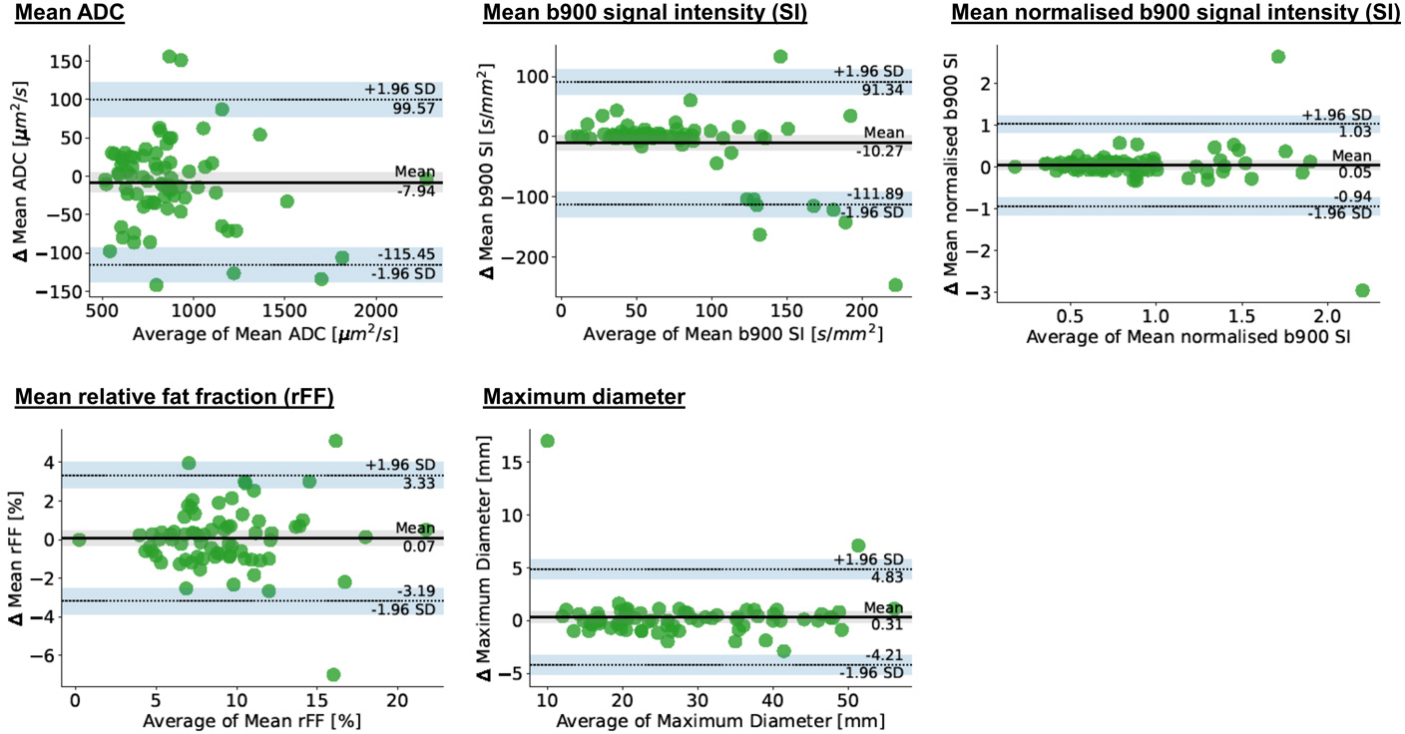
Bland-Altman plots for individual target lesion measurements, Δ – change in parameter.

Medians of measurements across all individual target lesions were ADC 812 µm^2^/s, b900 SI 61.5, normalised b900 SI 0.79, rFF% 8.9 and maximum lesion diameter 26 mm. Repeatability LoA and RCs of ADC, b900 SI, normalised b900 SI, rFF% and maximum target diameter measurements derived from individual target lesions are summarised in Table 2. Corresponding Bland-Altmann plots are shown in Figure 3.

**Table 2.** Individual target lesion measurement repeatability

Parameter	95% LoAs	RC	CoV
Apparent diffusion coefficient in µm^2^/s *Percentages*	−115;+100 *−13%;+11.5%*	108 *12.5%*	*4.5%*
B900 signal intensity in Units *Percentages*	−91;+112 *−122%;+149%*	103 *137%*	*49.5%*
Normalised B900 signal intensity *Percentages*	−0.94;+1.03 *−106%;+116%*	0.98 *110.5%*	*39.9%*
Relative fat-fraction in %	−3.2;+3.3	3.24	12.8
Maximum diameter in mm *Percentages*	−4.2;+4.8 *−15%;+17%*	4.5 *16%*	*5.8%*

LoA, limits of agreement; RC, repeatability coefficient.

*Changes of the parameter in relation to the average mean measurement value in italics*

Good repeatability was found for individual target lesion means of ADC, rFF% and maximum diameter. By comparison, individual target lesion means of b900 SI and normalised b900 SI showed poor repeatability.

### Per patient target lesion averages

Medians of patient target average measurements were ADC 833 µm^2^/s, b900 SI 58.9, normalised b900 SI 0.89, rFF% 9.1 and maximum diameter 26 mm.

**Figure 4.**
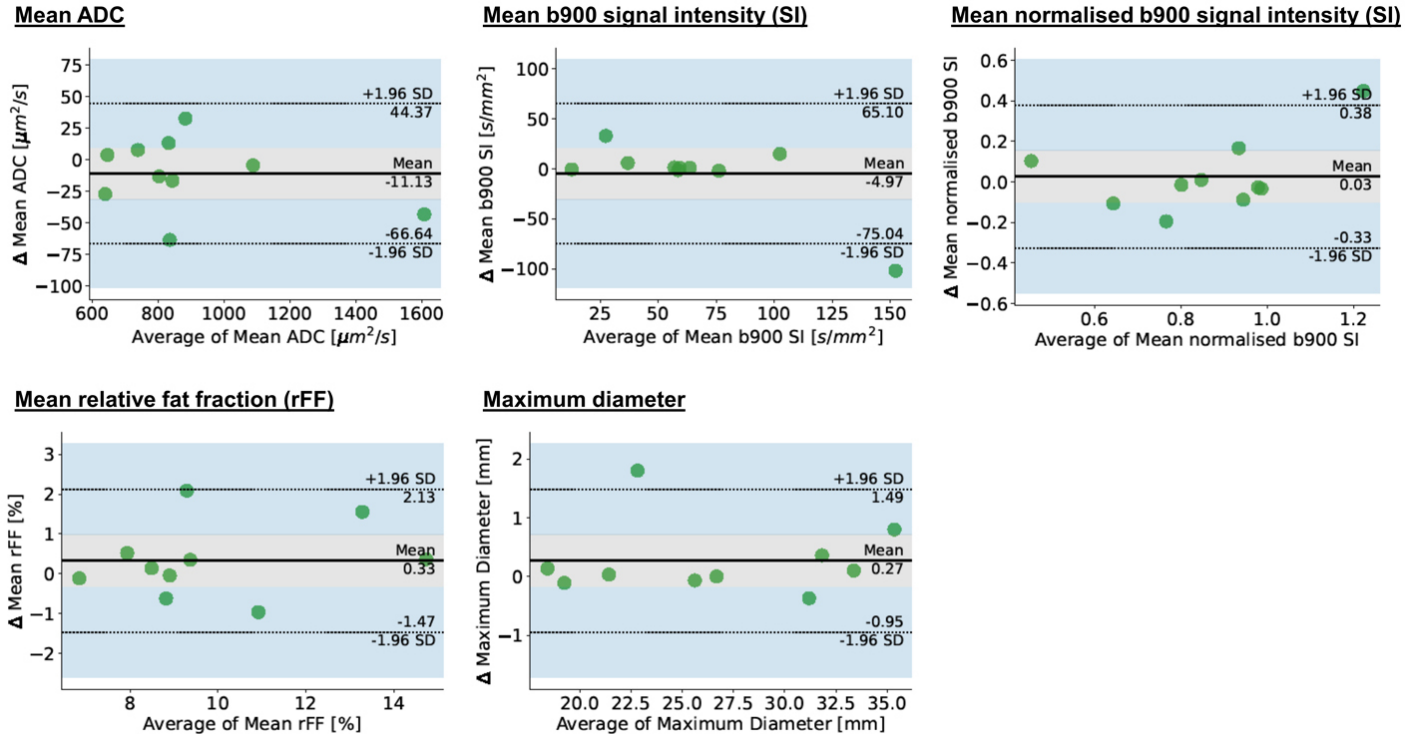
Bland-Altman plots for patient target lesion averages, Δ – change in parameter.

Repeatability LoA and RCs of average means of ADC, b900 SI, normalised b900 SI, rFF% and maximum diameter measurements across all target lesions per patient are summarised in Table 3. Corresponding Bland-Altmann plots are shown in Figure 4.

**Table 3.** Per patient target lesion average derived measurement repeatability

Parameter	95% LoAs	RC	CoV
Apparent diffusion coefficient in µm^2^/s *Percentages*	−67;+44 *−7.5%;+5%*	57 *6.5%*	*2.3%*
B900 signal intensity in Units *Percentages*	−75;+65 *−116%;+101%*	67 *104%*	*37.5%*
Normalised B900 signal intensity *Percentages*	−0.32;+0.38 *−38%;+44%*	0.34 *39.5%*	*14.3%*
Relative fat-fraction in %	−1.5;+2.1	1.82	6.7
Maximum diameter in mm *Percentages*	−0.9;+1.5 *−3.5%;+5.6%*	1.27 *4.8%*	*1.7%*

CoV, coefficient of variation; LoA, limits of agreement; RC, repeatability coefficient.

*Relative percentage changes of the parameter in relation to the average mean measurement value in italics*

Good repeatability was found for patient average means of ADC, rFF% and maximum diameter target measurements. Patient target averages of b900 SI and normalised b900 SI showed poor repeatability.

### Manual bone disease volume segmentation

**Figure 5.**
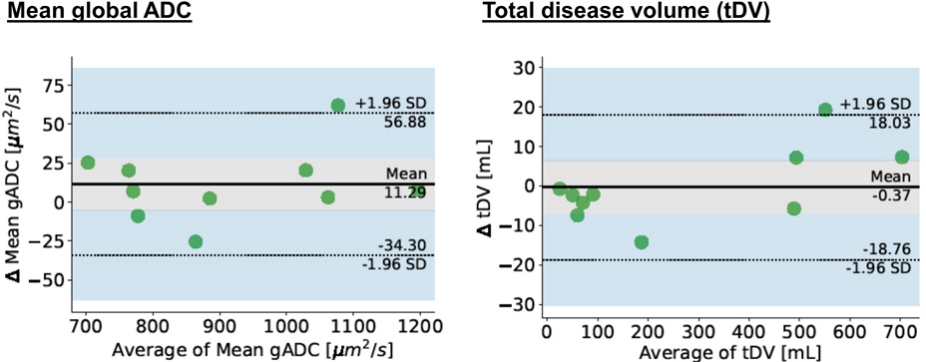
Bland-Altman plots for global ADC and total disease volume, Δ – change in parameter.

Across all patients median tDV was 139 ml and median gADC was 875 µm^2^/s. Repeatability parameters are shown in Table 4 and the corresponding Bland-Altmann plot in Figure 5. tDV and gADC showed good repeatability.

**Table 4.** Patient total disease volume derived measurement repeatability

Apparent diffusion coefficient	95% LoAs	RC	CoV
Mean in µm^2^/s *Percentages*	−34;+57 *−4%;+6.5%*	48.5 *5.3%*	1.9%
Total disease volume in µm^2^/s *Percentages*	−18.5;+18 *−6.9%;+6.6%*	17.5 *6.4%*	2.3%

CoV, coefficient of variation; LoA, limits of agreement; RC, repeatability coefficient.

*Relative percentage changes of the parameter in relation to the average mean measurement value in italics*

### Comparison of patient target lesion average ADC and global ADC

Paired analyses revealed no significant difference between the patient average target lesion mean ADC (891 ± 282 µm^2^/s) and gADC (912 ± 168 µm^2^/s, *p* = 0.386). Average target mean ADC ranged from 639 to 1607 µm^2^/s and gADC from 703 to 1196 µm^2^/s.

## Discussion

This study demonstrated good repeatability for means of individual targets as well as patient target average APC bone metastases’ ADC, rFF% and maximum diameter. Change in mean ADC > 12.5% and rFF% > 3.2 in individual lesions, mean ADC > 6.4% and rFF% > 1.8 in patient target averages and gADC>5.3% can be considered meaningful in a test-retest setting. Patient target lesion ROI average mean ADC was comparable to volumetric patient gADC, validating the feasibility and utility of pragmatic target measurements for assessment of APC bone metastases.

In a recent study, Elgendy et. al. described good ADC test-retest repeatability in 23 focal multiple myeloma lesions in 11 patients. The authors also included 24 sites of diffuse disease in their analyses and found ADC LoAs of −190.1 + 212.3 and a CoV of 14.5%.^22^ The lower CoV of 4.5% across our 73 individual target lesions may be attributed to using three consecutive slices for ADC measurements, thus improving repeatability compared with single slice ROI measurements. Moreover, the authors noted improved repeatability of measurements of focal lesions compared with diffuse disease.^22^ Another study including seven multiple myeloma patients showed an 8.3% RC.^27^ This is comparable to the 5.3% gADC RC reported in our study. Wennmann et. al. found a 16.2% repeatability CoV for iliac crest and sacral bone marrow measurements in patients with monoclonal plasma cell disorders, but did not report ADC measurements of focal lesions.^21^ Another study analysing healthy L5 and pelvic bone marrow of nine volunteers using ROI measurements demonstrated 14.8% ADC RC.^28^

The ADC repeatability in focal bone metastases has not been described to date. Nevertheless, APC mpWB-MRI interpretation guidelines suggest that *a* ≥ 25% bone metastasis’ ADC increase between two time points identifies therapy response.^4^ The 25% threshold corresponds to twice the demonstrated individual target 12.5% mean ADC RC. It is common practice in threshold-based decision-making to use twice the RC to confidently identify meaningful parameter change. Thus, our study provides evidence for the “METastasis Reporting and Data System for Prostate Cancer” recommendation on ADC interpretation in the context of therapy response.^4^ Consequently, our results suggest, that when comparing baseline with post-therapy examinations in an APC patient with bone-only disease, a 10 target-lesion average or total disease volume ADC change larger than 13% or 11%, respectively, indicate global patient response (applying the same practice of using twice the RC as response threshold). It needs to be noted, that within the same institution, follow-up examinations can be read by different radiologists. Subsequently, interrater variability will affect and contribute to ADC measurement variance. However, previous studies have demonstrated excellent interrater agreement of ADC measurements in metastatic bone disease and focal multiple myeloma lesions.^22,23,29^ As such, using twice the RC as a response threshold to indicate meaningful ADC change, should provide sufficient margin to compensate for interrater variability. By contrast, b900 SI and normalised b900 SI showed poor repeatability (RCs ≥ 39.6%). This was anticipated as DWI SI measurements lack the inherent normalisation of the ADC or rFF%. This study supports the suggestion to refrain from drawing conclusion from DWI SI measurements without advanced normalisation techniques.

rFF% is supplied in percentage values between 0 and 100. Based on our findings, an rFF% change in individual APC metastases > 3.2 and target average rFF% > 1.8 indicate meaningful change in a test-retest setting. This can help to identify therapy benefit in cases of rFF% increase between baseline to follow-up imaging during therapy. Excellent inter- and intrareader agreement has been shown for rFF% of focal multiple myeloma lesions, but previous test-retest repeatability analyses are lacking.^29^ A proton-density fat-fraction (PDFF) phantom study demonstrated 0.31–1.58% absolute PDFF RC supporting the good repeatability identified in our *in vivo* study.^30^ While ADC is the most promising single MRI biomarker for assessment of focal bone disease, with an continuously growing body of supporting literature, rFF% may have a supporting role to identify response with fatty marrow return. Additionally, rFF% change may provide decision support in equivocal cases.

T1w rFF% image derived maximum target diameter measurements showed good repeatability on individual lesion and patient level. An individual target maximum diameter change ≥4.5 mm can be assumed to be meaningful in a retest setting and indicates relevant change, such as worsening disease with increasing lesion size. This is almost identical to the 4.4 mm RC found in repeatability diameter measurements across 140 focal multiple myeloma lesions in 37 patients^31^ and supports the role of MRI in bone disease, enabling repeatable diameter measurements of disease labelled “non-measurable” by RECIST 1.1.^24^

tDV and gADC showed good repeatability (RC≤6.4%), further supporting their use as imaging biomarkers. Pragmatic average target ROI ADC measurements performed on PACS showed comparable repeatability. There was no significant value difference between patient target average mean ADC and gADC (*p* = 0.336). Consequently, pragmatic measurements of up to 10 targets can inform on APC patients’ metastatic bone disease status and may be utilised for staging of bone disease. Our findings can serve as a basis upon which evidence-based target response criteria incorporating mean ADC, rFF% and maximum diameter measurements for bone metastases (bone-RECIST) can be developed and validated. Bone metastases’ ADC and rFF% values reported in our study are in keeping with those found in the literature, suggesting general applicability of our findings in APC patients undergoing 1.5T WB-mpBMRI.^5,6,17^This study has limitations. First, only 10 patients (73 targets) were included. For the requirement of repeat scans, repeatability studies are time and labour intensive and usually include few patients. Second, the analysed dataset was acquired in 2013 on a Siemens Magnetoma Aera system. While this does not represent the latest 1.5T MRI scanner generation, it is currently in the vendor’s sale portfolio and likely representative for globally employed MRI hardware. This also applies to the used sequence parameters. Third, reproducibility was not assessed in this study. Knowledge on biomarker reproducibility is essential for intercentre comparison, but as they also require re-test examinations across different sites and/or hardware vendors, they are rarely performed.^32^ In follow-up scenarios with imaging performed on the same scanner using the same sequences, repeatability data can give substantial evidence to allow for in-centre comparisons. Finally, ROIs were not copied from original onto retest examination, but drawn manually. This desired scenario was chosen to reflect real life repeatability.

## Conclusion

In conclusion, individual as well target average APC bone metastases’ ADC, rFF% and maximum diameter, tDV and gADC show good repeatability, supporting their use as potential imaging response biomarkers for bone metastases.
